# Amniotic fluid-derived stem cells: potential factories of natural and mimetic strategies for congenital malformations

**DOI:** 10.21203/rs.3.rs-4325422/v1

**Published:** 2024-06-04

**Authors:** Cristiane S.R. Fonteles, John W. Steele, Daniel Ifeoluwa Idowu, Beck Burgelin, Richard H. Finnell, Bruna Corradetti

**Affiliations:** Universidade Federal do Ceara Faculdade de Farmacia Odontologia e Enfermagem; Baylor College of Medicine; Baylor College of Medicine; Baylor College of Medicine; Baylor College of Medicine; Baylor College of Medicine

**Keywords:** exosomes, mimetics, congenital malformations, mRNA therapeutics, ex vivo embryo culture

## Abstract

**Background:**

Mesenchymal stem cells (MSCs) from gestational tissues represent promising strategies for *in utero* treatment of congenital malformations, but plasticity and required high-risk surgical procedures limit their use. Here we propose natural exosomes (EXOs) isolated from amniotic fluid-MSCs (AF-MSCs), and their mimetic counterparts (MIMs), as valid, stable, and minimally invasive therapeutic alternatives.

**Methods:**

MIMs were generated from AF-MSCs by combining sequential filtration steps through filter membranes with different porosity and size exclusion chromatography columns. Physiochemical and molecular characterization was performed to compare them to EXOs released from the same number of cells. The possibility to exploit both formulations as mRNA-therapeutics was explored by evaluating cell uptake (using two different cell types, fibroblasts, and macrophages) and mRNA functionality overtime in an *in vitro* experimental setting as well as in an *ex vivo*, whole embryo culture using pregnant C57BL6 dams.

**Results:**

Molecular and physiochemical characterization showed no differences between EXOs and MIMs, with MIMs determining a 3-fold greater yield. MIMs delivered a more intense and prolonged expression of mRNA encoding for green fluorescent protein (GFP) in macrophages and fibroblasts. An *ex-vivo* whole embryo culture demonstrated that MIMs mainly accumulate at the level of the yolk sac, while EXOs reach the embryo.

**Conclusions:**

The present data confirms the potential application of EXOs for the prenatal repair of neural tube defects and proposes MIMs as prospective vehicles to prevent congenital malformations caused by *in utero* exposure to drugs.

## 1. INTRODUCTION

Neural tube defects (NTDs) are among the most severe and prevalent human congenital malformations, affecting on average 1.9 per 1000 live births (0.8–3.1 per 1000 live births) worldwide, and resulting in hundreds of thousands of associated deaths.^[1]^ Lack of NTD-prevention programs significantly increases the global burden caused by NTDs in low- and middle-income countries (LMIC), generating the highest prevalence of NTD-associated stillbirths in Asia and Africa.^[1]^ These defects are a costly medical burden, as correction of these anomalies generally requires multiple surgeries and long-term monitoring.^[2, 3]^ Prenatal surgical repair mediated by the application of regenerative strategies (i.e., biomaterials, stem cells or a combination of both) has been proposed to reduce the severity of these malformations, but these tend to be high-risk surgical procedures to both mother and infant.^[4–6]^As an example, despite evidence that prenatal surgery significantly improves clinical outcome for infants affected by spina bifida by reducing the need for ventriculoperitoneal shunt placement, motor function and mental development improvements, preterm labor, uterine dehiscence, neonatal death, and preterm birth still remain highly prevalent upon treatment.^[7, 8]^

Mesenchymal stem cell (MSC)-based approaches, especially those derived from gestational tissues (i.e., placental tissues, umbilical cord), have been widely studied as potential strategies to create an *in utero* pro-regenerative environment, due to the role they play in mediating embryo-maternal communication.^[9]^ Advantages in the use of these tissues over adult counterparts include the possibility to establish a cell-banking system as they can yield a great number of cells noninvasively and without posing unnecessarily complex ethical issues.^[10]^ Transamniotic therapy mediated by placental and amniotic fluid derived MSCs (AF-MSCs) has showed a protective effect for the treatment of fetal and neonatal congenital disorders.^[11]^ It is now widely established that MSCs act as trophic mediators, modulating the function of surrounding endogenous cells by releasing paracrine signals (growth factors, cytokines, chemokines, and extracellular vesicles (EVs).^[12, 13]^ MSC-derived EVs, including exosomes (EXOs; 50–130nm in size), are natural nanoparticles generated by double invagination of the plasma membrane and the formation of intracellular multivesicular bodies containing intraluminal vesicles.^[14]^ By maintaining parental physiochemical and molecular properties,^[15, 16]^ displaying inherent targeting capabilities and endogenous homing markers (which makes them able to cross biological barriers), EXOs are currently considered as promising diagnostic and therapeutic tools.^[17]^

In addition to exerting similar effects to those associated to the cells they are released by, EXOs have been proposed as natural delivery systems able to increase the efficiency and targeted specificity of therapeutics.^[18]^ Our laboratory has recently developed an efficient approach to utilize EXOs as reconfigurable systems for the delivery of a chemotherapeutic agent, doxorubicin (DOXO), for the treatment of advanced ovarian cancer.^[19]^ In addition, we established a platform based on a cell extrusion approach to increase (of about 3-fold) the production of exosomal therapeutics. The nanoparticles we obtained, called Immune Derived-exosome Mimetics (IDEM), are versatile nanoscopic therapeutics that retain the molecular features of EXOs isolated from the same number of monocytic cells with an increased structural stability. When loaded with DOXO, IDEM showed an incremental encapsulation efficiency (EE) compared to values reported in literature for naturally released EXOs,^[20]^ a marked release that guarantees an increased uptake by target cancer cells, in 2D and 3D culture systems, as well as a more effective cytotoxic and apoptotic effect of DOXO-loaded particles compared to the free drug.

In this study, we propose to generate EXOs and mimetics (MIMs) from amniotic fluid-derived MSCs (AF-MSCs) as potential regenerative tools to be used for the treatment (or prevention) of congenital malformations. Upon a comprehensive physiochemical and molecular characterization, the possibility to exploit both formulations as mRNA-therapeutics has been explored by evaluating cell uptake (using two different cell types, fibroblasts, and macrophages) and mRNA functionality overtime in an *in vitro* experimental setting as well as in an *ex vivo*, whole embryo culture. The latter was performed as a proof-of-concept system to determine differences in the biodistribution potential between natural and mimetic strategies.

## 2. MATERIALS AND METHODS

### 2.1 Cell culture

Amniotic fluid MSCs (AF-MSCs) were purchased from Celprogen and maintained using a Mesenchymal Stem Cell Growth kit (ATCC). Murine macrophages (J774 cell line) were purchased from ATCC and cultured in High Glucose-Dulbecco’s Modified Eagle Medium (HG-DMEM) (ThermoFisher Scientific) supplemented with 10% fetal bovine serum (FBS) (ThermoFisher Scientific), 1% L-glutamine and 100 U/ml Penicillin-Streptomycin (PS) solution (Sigma-Aldrich). Fibroblast (MRC-5 cell line) cultures were maintained in F12-DMEM (Gibco) supplemented with 15% heat-inactivated FBS, 1% L-glutamine and 100 U/ml PS solution (Sigma-Aldrich). Culture conditions were established at 37°C and 5% CO_2_.

### 2.2 Exosome and mimetics production

**Figure 1:**
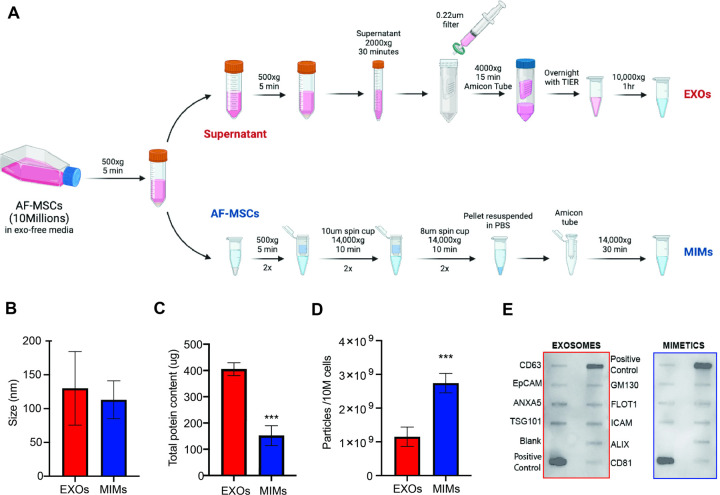
AF-MSC-derived mimetics production and characterization. **(A)** Schematics of mimetics (MIMs) production compared to naturally released exosomes (EXOs): MIM production occurs through filtered-membrane centrifugation steps while EXO extraction from culture media by using the Total Exosome Isolation Reagent (TEIR). Concentration (particle/ml) **(B)** and size **(C)** values for mimetics (MIMs, in red) and exosomes (EXOs, in blue) obtained by NTA (*n*=10). Statistically significant differences (p*** < 0.001) were observed between the two particle type formulations in terms of yield starting from the same number of AF-MSCs (10 millions/batch). **(D)** Total protein content (expressed in mg) shows a reduction in MIMs compared to EXOs (n=3, p*** < 0.001). **(E)** Protein array displays comparable qualitative molecular profiles between MIMs and EXOs.

AF-MSCs (10 × 10^6^) at passage 3 (P3) were grown in standard media supplemented with Exo-free FBS for 24 hrs. Media and cells were collected and processed following previously established protocols to isolate naturally released EXOs and to produce MIMs, respectively [19]. Exosomes were isolated by subjecting media to a series of centrifugations required to remove the cellular component (500 x g for 5 min) and any debris (2000 x g for 30 min). The remaining supernatant was passed through 0.22 mm PES membrane filter (CellTreat) and then concentrated using 10KDa Amicon ultra centrifugal filters (Millipore). Total exosome isolation reagent (Invitrogen) was then added in a 1:1 ratio to the volume obtained after the Amicon-based concentration process. The solution was mixed by vortexing for 30 seconds and incubated overnight at 4°C. The next day, the sample was centrifuged at 10,000 X g for 1 hour at 4°C. The concentrated solution was centrifuged at 10,000 X g for 1 h at 4°C, and the pellet was resuspended in 0.22mm filtered PBS. Mimetics (MIM) were produced by deconstructing and reconstructing cells through porous membranes of decreasing size. Briefly, AF-MSCs were harvested and washed twice in PBS. The PBS-resuspended pellet was then filtered through 10mm-filter Pierce^™^ spin cups (ThermoFisher) and centrifuged at 14,000 X g for 10 min at 4°C. The pelleted flow-through was resuspended in PBS and the same process repeated. Consequently, the pellet was passed through 8 mm filters (Merck-Millipore) with the same centrifuge settings as before. The pellet was finally resuspended in 150 µL of 0.22 µm-filtered PBS and run through G-50 Sephadex high-capacity spin columns (Sigma Aldrich) for further purification of the solution. Figure 1A shows the steps required for MIM production. MIM were also generated utilizing frozen AF-MSCs (F-MIMs) to evaluate the feasibility of this approach without the need to manipulating fresh cells. Exosomes and mimetics were stored at −80°C or immediately used for downstream applications.

### 2.3 AF-MSC derived exosomes and mimetics characterization

#### Nanoparticles Tracking Analysis (NTA).

Exosome and mimetic samples were analyzed according to the MISEV2018 Minimal information for studies of EVs.^[21]^ The NS300 Nanosight System (Malvern) was used to determine size and concentration. A 100X dilution in PBS was prepared for each sample. Briefly, 5 videos of 60 seconds each were recorded for each sample, and the threshold was kept constant at 5. Measurements for both formulations were repeated n = 10 times to obtain statistically robust data.

#### Evaluation of exosomal markers.

Total protein concentration in exosomes and mimetics was determined by using Pierce BCA Protein Assay (Pierce) and the presence of 8 specific exosomal markers (CD63, EpCAM, ANXA5, TSG101, GM130, FLOT1, ICAM, ALIX and CD81) was assessed using Exo-Check^™^ Exosome Antibody Array (System Biosciences) and following manufacturer’s instructions.

### 2.4 mRNA encapsulation and encapsulation efficiency (EE%) assessment

Complete N1-methylpseudouridine-substituted mRNA used in this study was purchased from the RNA Therapeutic Core at Houston Methodist Research Institute (Houston, USA). As a proof-of-concept study, mRNA encoding for the nuclear green fluorescent protein was encapsulated. GFP-mRNA loading within exosomes and mimetics was achieved using Exo-Fect^™^ Exosome Transfection Kit (System Biosciences) following manufacturer’s instructions. Unencapsulated mRNA was removed using an Exosome Spin Column (Invitrogen). After loading, the encapsulation efficiency (EE%) was measured by Quant-it^™^ RiboGreen RNA Assay Kit (Invitrogen), which allows for a sensitive detection of RNA in the range of 1–200 ng. 0.1% of Triton-X-100 was added to the samples for 10 min at RT to determine differences between free and encapsulated mRNA. The concentration of mRNA in both exosomes and mimetics was determined by exciting samples at 485 nm and assessing emission values at 530 nm fluorescence microplate reader (Synergy H4 Hybrid Plate Reader, Biotek).

### 2.5 Exosome and mimetic-mediated cellular uptake and GFP-mRNA expression

To evaluate exosome- and mimetic-mediated cell uptake by human fetal lung fibroblasts (MRC-5) and mouse macrophages (J774), 1×10^4^/cm^2^ MRC-5 and J774 cells were seeded into 6-well plates and allowed to adhere overnight. The next day, exosomes and mimetics were stained with 5 µM Vybrant DiD dye (ThermoFisher) in a final volume of 500 µl for 10 min at 37°C. Washes were performed by ultracentrifuging mixture at 40,000xg for 1 hr at 4°C. The pellet was resuspended in 1 ml of 0.22 µm filtered PBS (Gibco) and exosome spin columns (MW3000, Invitrogen) were used to remove any unbound dye. DiD stained exosomes and mimetics (at the concentration of 1×10^8^) were added to each well. Cellular uptake was quantitatively evaluated at different time points (4, 8, 12 hrs) by fluorescence microscopy. The efficiency of exosomes and mimetics in protecting mRNA while keeping it functional, MRC-5 and J774 were exposed to mRNA-loaded particles for 24, 48 and 72 hrs. At the end of each incubation cells were processed for flow cytometry. The same approach was followed to test the efficacy of F-MIMs as delivery systems.

### 2.5 Fluorescence microscopy

At 3 different time points (4, 8, and 12 hrs) cells were washed twice in pre-warmed PBS at pH 7.4, fixed in 4% paraformaldehyde (PFA) for 10 min at RT and washed three times in PBS for 5 min/wash. After washing, the samples were visualized and imaged with a Nikon microscope.

### 2.6 Flow cytometry

Flow cytometry was used to quantify the percentage of GFP-expressing cells or cells containing DiD-labelled exosomes and mimetics. At each time point, J774 and MRC-5 cells were analyzed for the presence of DiD at the excitation of 480nm and emission at 590nm, while GFP expressing cells was identified using the 488 nm excitation laser. Mean fluorescence intensity (MFI) was accounted for to evaluate changes in the levels of GFP expression overtime. Ten thousand events per sample were acquired with a BD LSR Fortessa^™^ flow cytometer, and the FCS/SSC parameters were used to gate cells. .fcs files were analyzed using Flowjo software.

### 2.7 *Ex vivo* whole embryo culture

*Ex vivo* studies were conducted following the approved protocol AN-7618 established by Baylor College of Medicine’s Institutional Animal Care and Use Committee (IACUC) in accordance with the guidelines of the Animal Welfare Act and the Guide for the Care and Use of Laboratory Animals, as well as adhering to the ARRIVE guidelines 2.0. Animal protocol title “Intervention strategies for non-folate responsive neural tube defects”, approval date 11/17/2023. Three Pregnant C57BL6 dams (2–4 months old) were euthanized on gestational day 8.5 according to the IACUC-approved “Euthanasia in rodents policy” and the CCM policy of “Euthanasia of adults and neonatal rodents in Smartbox units” by using automated CO_2_ euthanasia chambers. The uterus was resected and placed in warm HEPES-buffered Tyrode’s Solution (Thermo Scientific) for dissection. Using forceps, the uterus was peeled away from the conceptus, and the decidual capsule and Reichardt’s membrane were carefully removed to leave the embryo and yolk sac intact. Embryos (n = 7/experimental group) were randomly assigned to two groups (mimetics and exosomes) and were cultured in 100% immediately centrifuged rat serum (Envigo) containing 10^8^ exosomes or mimetics for 24 hours while rotating in roller bottles at 37.5°C. Prior to culture, the rat serum was equilibrated with a 5% O_2_/5% CO_2_ gas mixture (AirGas) by gently blowing the gas mixture over the surface of the serum within the roller bottle for approximately 60 seconds. Each roller bottle contained 4 mL of serum and no more than 4 embryos were cultured per bottle. After 24 hours, embryos were removed from the culture bottles, washed briefly in PBS, and the embryo was then separated from the yolk sac. Localization of exosomes or mimetics was assessed qualitatively by confocal microscopy as reported below.

### 2.8 Confocal microscopy

Embryos and yolk sacs exposed to exosomes or mimetics were fixed on ice for 30 minutes in 4% PFA. They were then washed twice in PBS before being placed in 1mL of blocking buffer (1% BSA in PBS) in a microcentrifuge tube. The microfuge tube was pre-incubated with blocking buffer 1 hour prior to prevent the embryos and yolk sacs from sticking to the walls of the tube. The embryos and yolk sacs were incubated in blocking buffer for 1 h while rotating at room temperature. Hoechst (1µg/mL) and Phalloidin-iFlour 488 or Phalloidin-iFlour 594 (1:1000) (Abcam, ab176753/ab176757) were added to the blocking buffer and the embryos and yolk sacs were incubated overnight while rotating at 4°C. They were then washed in 3, 1mL volumes of PBS (1 hour per wash while rotating at room temperature). Whole embryos and yolk sacs were then imaged using a CSU-W1 Spinning Disk Confocal system (Nikon Center of Excellence, CPEH, Baylor College of Medicine).

### 2.9 Statistical analysis

Data was initially reported as mean, standard deviation, standard error, median, minimum and maximum considering two different categories or groups of exosomes produced by MIMs versus standard extraction EXOs. In a sequence, normality was tested. Number (yield) and diameter (size in nm) were compared between groups using Independent-Samples Mann-Whitney U Test, and differences were considered significant when p < 0.05. For protein quantification, mRNA encapsulation efficiency, MFI, a two-tailed Student’s t-test was performed. All graphs show average values and standard deviation.

## 3. RESULTS

### 3.1 Mimetics display exosomal size and molecular moieties

Nanoparticle tracking analysis (NTA) was used to determine size and concentration of the two formulations. Starting from the same number of AF-MSCs (1×10^6^), the optimized procedure allowed to produce 2.74×10^10^ mimetics compared to 1.15×10^9^ exosomes obtained following standard protocols for the isolation of natural exosomes from culture media (Fig. 1B), showing a 2.38-fold increase compared to natural counterparts (p < 0.001). The same yield is not obtained when mimetics are produced from frozen cells (Supplementary Fig. 1A). MIMs present an average size of 113 ± 28 nm while EXOs 130 ± 54 nm, respectively (Fig. 1C). No differences in terms of size were found between MIMs and F-MIMs (113 ± 28 vs 105 ± 9.09, respectively) (Supplementary Fig. 1B). Total proteins evaluated showed a reduction in MIMs compared to EXOs (Fig. 1D), although the qualitative analysis confirmed the presence of specific exosomal markers (Cd63, EpCAM, ANXA5, TSG101, CD81, ALIX, ICAM, FLOT1, GM130) with no differences between the two particle types (Fig. 1D). Characterization data were corroborated by scanning electron microscopy to provide morphological information about AF-derived mimetics compared to natural counterparts (Fig. 1E).

### 3.2 mRNA-GFP delivered through mimetics maintain expression overtime in different cell types

**Figure 2:**
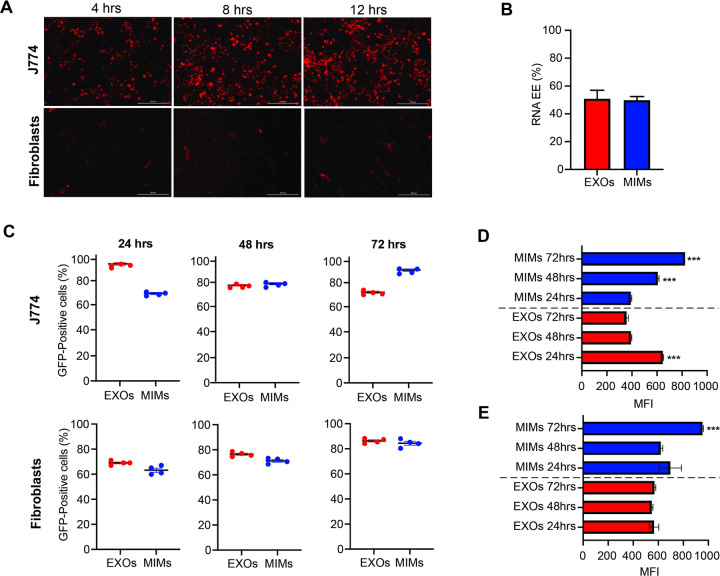
Differential cellular uptake of fluorescently labelled and mRNA-loaded mimetics. **(A)** Fluorescence microscope images showing DiD-labelled mimetics taken up by two cell lines, murine macrophages (J774) and human fibroblasts (MRC-5) at early time points (4, 8 and 12 hrs). (Magnification: 10X, Scale bar:100 µm). **(B)** Graph showing mRNA-GFP encapsulation efficiency (EE) in MIMs and EXOs as revealed by Quant-it^™^ RiboGreen RNA Assay Kit. (n=3). **(C)** Time-dependent appearance of GFP fluorescence expressed as percentage of GFP-positive cells in macrophages and fibroblasts observed by flow cytometry after 24 h, 48 h and 72 h post transfection with MIMs. EXOs are used for comparison. (Data represented as mean ± SD, n=4). Quantification of changes in the mean fluorescent intensity detected in macrophages **(D)** and fibroblasts **(E)** at each time point. Untreated cells used as baseline. (n=4, p*** < 0.001).

The cell uptake of DiD-labelled MIMs was tested on human fibroblasts (MCF-5) and murine macrophages (J774). There was a differential cell uptake of MIMs, with J774 cells incorporating more particles at early time points than their fibroblastic counterparts (Fig. 2A). Encapsulation efficiency for mRNA was found comparable between MIMs and EXOs, being assessed around 49.79 ± 2.61 and 50.87 ± 6.11, respectively (Fig. 2B). After assessing mRNA EE, the expression of the mRNA encoding for GFP delivered through MIMs was quantitatively evaluated on fibroblasts and macrophages at 24, 48, and 72 hrs (Fig. 2C). Flow cytometry data show GFP-mRNA loaded MIMs and EXOs display a different trend as mRNA mediators when administered to J774 cells. In particular, the percentage of GFP-positive cells increases overtime when delivered by MIMs, with the highest expressions levels being reached at 72 hrs (up to 90%). However, in the EXO group, a slight reduction in the number of positive cells is observed overtime, with 92% of GFP-positive cells being found at 24 hrs. A similar trend between MIMs and EXOs is observed when administered to fibroblasts. In this case, the percentage of GFP-positive cells is assessed around 68 ± 2.58 and 63.22 ± 3.33 for EXOs and MIMs at 24 hrs and increases for both treatments up to 86.32 ± 1.81 and 84.42 ± 3.14 at 72 hrs, respectively. Accordingly, the MFI associated with GFP expression was found to increase overtime in J774 cells treated with MIMs, with values recorded at 48 and 72 hrs (602.75 ± 10.91 and 832.25 ± 12, respectively, being statistically highly significant (P < 0.01) compared to their EXO counterparts where decreasing values were found (391.5 ± 7.5 and 356.25 ± 15.68, respectively (Fig. 2D). On the other hand, fibroblasts uptake of mRNA mediated by MIMs showed a statistically significant increase in the MFI only at 72 hrs, compared to EXOs (952.25 ± 8.01 vs 568.75 ± 11.44) (Fig. 2E). When F-MIMs were administered to cells, differential uptake patterns were observed depending on the cell type as well as on the preparation method. F-MIMs are easily taken up by J774 cells although the number of GFP-positive cells and the MFI, fade overtime compared to freshly prepared MIMs (Supplementary Fig. 1C) shows that. Comparable trends between MIMs from fresh and frozen MSCs, were found upon administration to fibroblasts at 24 hrs and 72 hrs, although it was found doubled at 48 hrs for F-MIMs and only 24 hrs later for fresh MIMs.

### 3.3 Mimetic and Exosome biodistribution differs in *ex vivo* whole embryo culture

**Figure 3:**
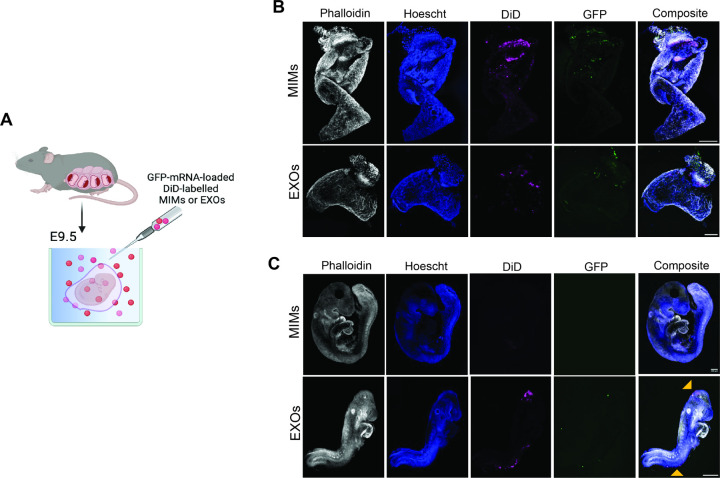
Mimetics and exosome biodistribution in whole embryo culture. **(A)** Schematic representation of whole embryo culture established to define mimetics (MIMs) and exosomes (EXOs) distribution *ex vivo*. Embryos were isolated from pregnant dams and cultured in presence of MIMs or EXOs (1×10^8^) administered in culture media for 24 hrs. Confocal microscopy images showing explanted yolk sac **(B)** and embryos **(C)** upon exposure to DiD-labelled (red), mRNA-GFP-loaded (green) MIMs or EXOs. Phalloidin (gray) and Hoechst (blue) were used to counterstain actin filaments and DNA, respectively. Scale bars: 500mm.

The potential biodistribution of MIMs as a potential therapeutic strategy for the treatment of congenital malformations was determined by using standard embryo cultures. Murine embryos at E9.5 were cultured for 24 hrs in the presence of DiD-labelled, GFP-mRNA-loaded MIMs or EXOs (Fig. 3A). At the end of the incubation period the yolk sac was dissected from the embryos and both components were observed using confocal microscopy, revealing the presence of DiD signal within the yolk sac upon exposure to both formulations (Fig. 3B). Signal associated to the expression of mRNA encoding for GFP was colocalized with the presence of MIMs and EXOs. However, while EXOs were found to be able to reach the embryos, as demonstrated by the expression of the DiD signal and GFP expression (although to limited extents), no signal was found upon exposure to MIMs (Fig. 3C). EXOs were localized for the most part in the cranial and ventral regions.

## 4. DISCUSSION

Tissue engineering approaches (including their combination with bioactive cells) represent promising treatment venues to repair structural birth defects.^[22–24]^ Yet, the application of these technological tools are still invasive and only limited advancements have been made in these clinical scenarios.^[25]^ The stem cell therapy alternative, mainly involving stem cells isolated from gestational tissues, have been reported to improve animal survival and guarantee *in utero* tissue repair in genetically and mechanically induced spina bifida.^[11, 26–28]^ Although promising, the inherent risks and hindrances of cell therapy are many and include but are not limited to issues with scalability, cell differentiation abilities and aging, bystander effect which reduces cell potency, number of cells reaching target sites, and therapeutic outcome.^[29]^

With this work, we propose nanotherapeutics derived from AF-MSCs as minimally invasive strategies for the prenatal repair of congenital anomalies. While EXOs from AF-MSCs have already been reported to retain parental cell molecular moieties and exert protective and regenerative potential,^[30, 31]^ here we compare them to those associated to exosome mimetics produced from AF-MSCs - obtained through a recently established process^[19]^ - as natural RNA therapeutics. Our data demonstrate that while both strategies can be used as scalable drug delivery systems, with the potential to be tailored for individual clinical applications. Production of MIMs yields a 2.38-fold greater concentration than natural EXOs isolated from the same number of source cells (fresh AF-MSCs, 1×10^6^). This trend is in line with previously acquired data, showing a 2–48 fold increase in the production of MIMs obtained from immune cells (monocytic cell line, called IDEM) through the same process compared to natural EXOs.^[19]^ MIMs also closely resemble their natural counterparts in terms of size, with a diameter (113 ± 28 nm) which falls within the expected range for natural EXOs,^[21, 32]^ although smaller when compared to IDEMs, and the expression of a similar cohort of proteins (Cd63, EpCAM, ANXA5, TSG101, CD81, ALIX, ICAM, FLOT1, GM130). Recently, Sayyed et al. produced cell-derived nanovesicles from human adipose-derived-MSCs by cell extrusion with a mean diameter of 177.3 ± 2 nm and a yield of 1×10^10^ from 1×10^6^ cells, surpassing the size produced in the current data, but showing a lower yield than was obtained in the present work.^[33]^ On the other hand, Zhang et al. reported a mean diameter of mimetic nanovesicles of 126.9 ± 3.0 nm, with a yield of 16 × 10^9^ particles from 1×10^6^ cells, and a total protein concentration of 122.8 µg per batch.^[34]^ Their yield and protein content were 20-fold higher than what they observed for natural EXOs. In our study, the yield of EXOs isolated from the same number of cells as MIM was greater than the ones reported by these authors, but an overall similar protein concentration was noted. Mimetics production from MSCs by Lu et al. generated EVs with a peak diameter mostly between 100–200 nm and a yield of approximately 1.6 × 10^6^.^[35]^ It should be borne in mind that the diameter and composition of naturally secreted EXOs vary according to cell type and physiologic status, and environmental conditions, such as extracellular stimuli they are exposed to (including culture media and oxygen levels).^[36]^ Also, techniques used for EXO extraction, including variations in centrifugation protocols, type of rotor and g-force are aspects that play an important role in magnifying the yield, purity, protein content, and size of these EVs.^[37, 38]^ Such variables should also be pondered in the production of MIMs and may explain differences encountered between the present results (in terms of yield, size and protein content) and data reported by others.^[33–35]^

Although challenging, mRNA encapsulation into EXOs represents a promising therapeutic strategy to various conditions, allowing for a more precise and ample control of protein expression than gene replacement therapy.^[39]^ In this study, we achieved a good RNA encapsulation efficiency with EXOs and MIMs. Moreover, GFP-mRNA loaded within DiD-labeled MIMs and EXOs was efficiently delivered and expressed by two different cell lines, demonstrating the marked ability of MIMs to preserve mRNA functionality. Overtime, MIMs led to greater percentage of GFP-positive macrophages and fibroblasts. Nonetheless, EXOs behaved differently within these cell lines with time, and the percentage of GFP-positive macrophages was higher in the initial 24 hours but reduced overtime, whereas the pattern of GFP expression by fibroblasts was much the same as observed in the presence of MIMs endowed with GFP-mRNA. Thus, the RNA-cargo loading expressed differential uptake, varying according to target cells (macrophages or fibroblasts) and nanoparticle types (MIMs or EXOs), and these differences translated into a more prolonged expression of mRNA delivered by MIMs. The consistent and increasing expression of GFP-mRNA delivered by MIMs endorses its more than adequate advantages over natural EXOs for encapsulation and delivery of mRNA mediated therapy.

We also evaluated the feasibility of using frozen AF-MSCs to generate MIMs (F-MIMs), to simplify the procedure by avoiding manipulation of fresh cells. Frozen cells produced a lower yield compared to fresh ones, but their diameter did not differ significantly, although the range of variation in the diameter of F-MIMs (min, 97.91, max: 119.01, 105 ± 9.09) was smaller than what was observed for MIMs (min: 85, max: 141, 113 ± 28). Cellular uptake evaluation demonstrated a reduction in the expression of GFP-positive macrophages over time, while a similar pattern of increased fibroblasts’ GFP expression was observed for MIM and F-MIM at 24 and 72 hrs, suggesting cryopreservation does not fully prevent the onset of apoptosis, impacting on cell recovery, which may explain the lower yield of F-MIMs and the differences observed when frozen cells were used.^[40]^

To our knowledge, the present study demonstrates for the first time the plausibility of applying the technology of MIMs and EXOs as a potential therapeutic strategy for congenital malformations. This aspect of the study was demonstrated by using *ex-vivo* whole embryo culture. Our data showed that embryos explanted at E9.5 and cultured in a “soup” of DiD-labelled, RNA-loaded EXOs or MIMs for 24 hrs, display the presence of a colocalization of signals at the level of the yolk sac where they expressed encapsulated GFP-mRNA. Importantly, no differences were found between the two formulations in support of embryo growth. Yet only EXOs reached the embryo. While the accumulation of MIMs at the level of the yolk sac deserves a more detailed evaluation on the molecular mechanisms detaining them from crossing the placenta, data obtained here suggest their potential role as reconfigurable drug delivery tools to prevent the teratogenicity caused by maternal intake of drugs known to be toxic for the fetus, such as various anti-seizure medications which remain a hurdle in the treatment of pregnant women with seizure disorders,^[41–43]^ and other non-epileptic conditions.^[44]^ In these instances, the chronic use of such substances is usually warranted to obtain adequate seizure control during pregnancy, raising serious concerns for pregnant women and those in childbearing age.^[45]^ Malformations caused by these drugs are frequently severe and include NTDs, congenital cardiac malformations and craniofacial malformations.^[46]^

On the other hand, since EXOs reach embryonic tissues and are primarily expressed in its cranial and ventral portions, they may be best suited for the prenatal repair of NTDs and other birth defects by loading these nanovesicles with cell-specific cargoes such as proteins, lipids, and nucleic acids.^[47]^ This is not to say that despite the lack of direct contact with the embryo, MIM may still play a role in the delivery of such molecules and be used in prenatal regenerative medicine through targeted delivery of genetic material to cells at the yolk sac by crosstalk and intercellular communication, due to its role in embryonic development.^[48, 49]^ Further studies are warranted to best understand these mechanisms.

## 5. Conclusions

The heterogenous nature of naturally secreted EXOs requires a complex and more time-consuming extraction rendering a significantly lower yield compared to MIMs and limiting their use for clinical application. Lack of standardized protocols imposes limitations when comparing results of mimetics reported by others, as well as in the RNA transfection system used to encapsulate mRNA into MIMs and/or EXOs. Although this type of system is considered a convenient method,^[39]^ efficiency of encapsulation remained within a 50% margin. The loading of EXOs and mimetic counterparts with mRNA remains a challenge to be overcome in future studies.^[50]^ The present data proposes MIMs as a promising strategy for high-throughput applications representing a better prospect for future clinical use as vehicles to reduce the incidence of congenital malformations secondary to *in utero* exposure to antiseizure medications and confirms the potential application of EXOs as minimally invasive strategies able to reduce the severity of NTD-associated aberrations for prenatal repair. Translational research utilizing these strategies is warranted to better comprehend the impact and extent of the present findings for clinical applications.

## Supplementary Material

Supplement 1

## Data Availability

Not applicable.

## Abbreviations

AF: amniotic fluid
ALIX: ALG-2-interacting protein X
ANXA5: Annexin A5
BSA: bovine serum albumin
DiD: DiIC18(5); 1,1′-dioctadecyl-3,3,3′,3′- tetramethylindodicarbocyanine, 4-chlorobenzenesulfonate salt
DMEM: dulbecco’s modified eagle medium
DOXO: doxorubicin
EE: encapsulation efficiency
EPCAM: epithelial cell adhesion molecule
EV: extracellular vesicles
EXO: exosomes
FCS/SSC: forward scatter/side scatter
FLOT-2: Flotillin 2
F-MIM: MIMs from frozen cells
GFP: green fluorescent protein
HEPES: 2-[4-(2-hydroxyethyl)piperazin-1-yl] ethanesulfonic acid
HG-DMEM: high-glucose dulbecco’s modified eagle medium
ICAM: Intercellular Adhesion Molecule 1
IDEM: immune derived exosome mimetics
MFI: mean fluorescence intensity
MIM: mimetics
mRNA: messenger ribonucleic acid
MSC: mesenchymal stem cells
NTA: nanoparticle tracking analysis
NTD: neural tube defects
P3: passage 3
PBS: phosphate-buffered saline
PFA: paraformaldehyde
PS: penicillin/streptomycin
RNA: ribonucleic acid
TSG101: tumor susceptibility gene 101
