# Supplementary material for: Amniotic fluid-derived stem cells: potential factories of natural and mimetic strategies for congenital malformations
Source: Res Sq. 2024 Jun 4:rs.3.rs-4325422. Preprint. [Version 1] doi: 10.21203/rs.3.rs-4325422/v1 (PMC11177991; doi:10.21203/rs.3.rs-4325422/v1)
Supplement: Supplement 1 [file NIHPPrs4325422v1-supplement-1.pdf]

Supplementary Files

This is a list of supplementary files associated with this preprint. Click to download.

- SupplementaryFig.1.tif
